# SARS-CoV-2 Reinfection and Risk of Multisystem Inflammatory Syndrome in Children: A Case–control Study

**DOI:** 10.1097/INF.0000000000005278

**Published:** 2026-05-20

**Authors:** Noémie Schiever, Lea Lenglart, Corinne Levy, Pierre-Alex Crisinel, Arnaud G. L’Huillier, Johan N. Siebert, Margherita Plebani, Yves Fougere, Alain Lefevre-Utile, Alexis Rybak, Eloise Burdet, Axelle Dupont, Olivier Brissaud, Jeanne Bordet, Lucas Percheron, Camille Brehin, Noémie Vanel, Caroline Ovaert, Alexandre Belot, Aurélie Morand, Julie Toubiana, Marion Grimaud, Stéphane Dauger, Julien Baleine, Stéphane Bechet, Alain Burtscher, Alain Wollner, Robert Cohen, Pierre-Louis Leger, Caroline Galeotti, Romain Basmaci, Camille Aupiais, Naïm Ouldali, François Angoulvant

**Affiliations:** From the * Service of Pediatrics, Department Women-Mother-Child, Lausanne University Hospital and University of Lausanne, Lausanne, Switzerland; † Pediatric Emergency Department, Robert Debré University Hospital, Assistance Publique-Hôpitaux de Paris, Paris Cité University, Paris, France; ‡ Paris Cité University, INSERM UMR 1137, Infection, Antimicrobials, Modelling, Evolution (IAME), Paris, France; § ACTIV, Association Clinique et Thérapeutique Infantile du Val-de-Marne, Créteil, France; ¶ Assistance Publique-Hôpitaux de Paris, Department of general pediatrics, pediatric infectious disease and internal medicine, Robert Debré university hospital, Université de Paris, Paris, France; ∥ Clinical Research Center (CRC), Centre Hospitalier Intercommunal de Créteil, Créteil, France; ** Pediatric Infectious Diseases and Vaccinology Unit, Service of Pediatrics, Department Women-Mother-Child, Lausanne University Hospital and University of Lausanne, Lausanne, Switzerland; †† Pediatric Infectious Diseases Unit, Department of Women, Child and Adolescent Medicine, Geneva University Hospitals and Faculty of Medicine, Geneva, Switzerland; ‡‡ Department of Pediatric Emergency Medicine, Geneva Children’s Hospital, Geneva University Hospitals, Geneva, Switzerland; §§ French Pediatric infectious Disease Group (GPIP), Paris, France; ¶¶ Department of Epidemiology and Clinical Research of Bichat Hospital, Bichat Hospital- Claude-Bernard, AP-HP, Paris, Île-de-France, France; ∥∥ Pediatric Intensive Care Unit, Children’s Hospital, Bordeaux University Hospital, Bordeaux, France; *** Strasbourg University Hospital, Pediatric cardiologist unit and Pediatric Intensive Care Unit, Hautepierre University Hospital, Strasbourg, France; ††† Purpan University Hospital, Pediatric Nephrology Department, Children Hospital, Toulouse, France; ‡‡‡ Purpan University Hospital, Pediatric Emergency Department, Children Hospital, Toulouse, France; §§§ Assistance Publique - Hôpitaux de Marseille, Department of Pediatric Anesthesia and Intensive Care, Timone Children Hospital, Aix-Marseille University, Marseille, France; ¶¶¶ Assistance Publique - Hôpitaux de Marseille, Pediatric and Congenital Cardiology Department, Timone Hospital Marseille, University Hospital, Marseille, France; ∥∥∥ INSERM, Marseille Medical Genetics, UMR 1251, Aix Marseille University, Marseille, France; **** Hospices Civils de Lyon, Pediatric Intensive Care Unit, Women-Mother-Child Hospital, University of Lyon, Bron, France; †††† EA 7426 “Pathophysiology of Injury-Induced Immunosuppression”, University Claude Bernard Lyon 1, Hospices Civils of Lyon, Lyon, France; ‡‡‡‡ Aix-Marseille University, Faculty of Medicine, Marseille, France; Department of General Pediatrics, University Hospital of La Timone, AP-HM, Marseille, France; §§§§ Assistance Publique – Hôpitaux de Paris, Department of General Pediatrics and Pediatric Infectious Diseases, Necker-Enfants-Malades University Hospital, Paris Cité University, Paris, France; ¶¶¶¶ Pasteur Institute, Biodiversity and Epidemiology of Bacterial Pathogens, Paris Cité University, Paris, France; ∥∥∥∥ Assistance Publique-Hôpitaux de Paris, Pediatric intensive care unit, Necker-Enfants Malades University Hospital, Paris Cité University, Paris, France; ***** Assistance Publique-Hôpitaux de Paris, Pediatric Intensive Care Unit, Robert Debré University Hospital, Paris Cité University, Paris, France; ††††† Pediatric Intensive Care Unit, Arnaud de Villeneuve University Hospital, Montpellier University, Montpellier, France; ‡‡‡‡‡ Association Française de Pédiatrie Ambulatoire (AFPA), Paris, France; §§§§§ Assistance Publique - Hôpitaux de Paris, Pediatric Intensive Care Unit, Armand Trousseau University Hospital, Sorbonne University, Paris, France; ¶¶¶¶¶ Department of Pediatric Rheumatology, Bicêtre University Hospital, Assistance Publique-Hôpitaux de Paris, Paris-Saclay University, Le Kremlin-Bicêtre, France; ∥∥∥∥∥ Assistance Publique - Hôpitaux de Paris, Pediatric Emergency Departement, Louis Mourier University Hospital, Colombes, France; ****** Department of General Pediatrics, Jean Verdier University Hospital, Assistance Publique-Hôpitaux de Paris, Bondy, France.

**Keywords:** pediatric multisystem inflammatory disease, COVID- 19-related, PIMS- TS, SARS-CoV- 2, pediatrics

## Abstract

**Background:**

Multisystem inflammatory syndrome in children (MIS-C) is a severe postinfectious complication of severe acute respiratory syndrome coronavirus 2 (SARS-CoV-2) infection, primarily affecting children 6–12 years of age. Although MIS-C was prominent during the early pandemic waves, recent studies suggest a declining incidence concurrent with increasing reinfection rates. The objective was to determine whether the risk of MIS-C differs following a primary SARS-CoV-2 infection versus reinfection.

**Methods:**

A retrospective, multicenter, international, matched case–control study was conducted from September 1, 2021, to April 30, 2022. MIS-C cases were recruited from 14 French and Swiss hospitals. Eligible cases included children younger than 18 years meeting the 2020 World Health Organization case MIS-C definition with laboratory evidence of SARS-CoV-2 infection. Cases were matched 1:1 by age, sex and epidemic period to controls with laboratory-confirmed acute SARS-CoV-2 infection. Vaccinated patients and those without consent were excluded. The primary outcome compared the association of reinfection with MIS-C versus acute infection using McNemar’s test to estimate matched odds ratios (ORs). Prespecified sensitivity analyses used conditional logistic regression.

**Results:**

The matched cohort included 180 MIS-C cases (median [IQR] age, 106 [62–136] months) and 180 controls (median [interquartile range] age, 72 [48–108] months). No MIS-C cases had a documented reinfection, whereas 3.9% (7/180) of controls did (McNemar χ^2^ = 5.142; *P* = 0.023; matched OR, 0.067; 95% confidence interval [0.004–1.167]; *P* = 0.064). Sensitivity analysis also suggested a lower odds of MIS-C after reinfection without reaching statistical significance (adjusted OR, 0.183; 95% [0.027–1.234]; *P* = 0.093).

**Conclusion:**

MIS-C occurred predominantly after first SARS-CoV-2 infections; while evidence for a lower risk following reinfection was suggestive but not conclusive.

Multisystem inflammatory syndrome in children (MIS-C) is a severe postinfectious inflammatory condition that appears 2–6 weeks after a severe acute respiratory syndrome coronavirus 2 (SARS-CoV-2) infection. It affects predominantly school-age children, with early cohorts reporting a median age around 8–9 years, frequent multisystem involvement (mucocutaneous, cardiovascular, gastrointestinal, hematologic, respiratory and nervous system), hospitalization in intensive care units, and approximately 2% of affected children die.^1–7^

The exact pathophysiology of MIS-C remains unclear. Immunophenotyping studies show that MIS-C is immunologically distinct from both acute pediatric coronavirus disease 2019 (COVID-19) and Kawasaki disease, consistent with a dysregulated, postinfectious immune response.^8^ MIS-C involves an expansion of activated T cell expressing the TRBV11-2 T-cell receptor.^9^ One hypothesis is that this signature is related to a superantigen-like motif in the SARS-CoV-2 spike protein, but this expansion, together with the MIS-C phenotype, was also recorded before the pandemic, suggesting that other triggers exist.^10,11^ Another hypothesis suggests that the inflammatory mechanism is driven by the formation of autoantibodies targeting cardiovascular, gastrointestinal and endothelial antigens.^3^ More recently, evidence has emerged supporting an autoimmune mechanism involving molecular mimicry, whereby autoantibodies generated against SARS-CoV-2 cross-react with SNX8, a human protein that regulates antiviral signaling pathways, potentially contributing to the hyperinflammatory response observed in MIS-C.^12^

Epidemiologically, the incidence and clinical severity of MIS-C have fallen across successive variant waves.^13,14^ Population-based and national surveillance analyses report markedly lower MIS-C incidence during Delta and especially Omicron compared with earlier periods, alongside evolving clinical phenotypes.^14,15^ At the same time, the share of SARS-CoV-2 cases that are reinfections increased from 1.5% during the Delta wave to 6.8% with Omicron.^16^

This observation allows for a comparison of the risk of MIS-C following SARS-CoV-2 reinfection versus primary infection. The research hypothesis is that the risk of MIS-C occurrence was higher after the first SARS-CoV-2 infection but decreased significantly during subsequent reinfections. Understanding whether prior SARS-CoV-2 reinfection influences the risk of subsequent MIS-C is critical for clarifying the postinfectious immune response and guiding pediatric infection control strategies.

The objective of this study was to determine whether the risk of MIS-C differs following a first SARS-CoV-2 infection compared with subsequent reinfections in children under 18 years.

## METHODS

### Study Design and Population

A retrospective, multicenter, international and matched case–control study was conducted.

This study compared children with MIS-C (cases) with children with acute SARS-CoV-2 infection (controls) over the same period from September 1, 2021, to April 30, 2022.

Cases were enrolled from France and Switzerland.

In France, cases were recruited in 10 tertiary hospitals within the Pandor Observatory Network (NCT04336956), a national network that recorded all patients hospitalized with severe forms of SARS-CoV-2-associated diseases, including MIS-C.^13,17^ In Switzerland, case patients were identified in 2 University hospitals (Lausanne and Geneva) using electronic medical records.

Eligibility for cases was age younger than 18 years and fulfillment of the 2020 World Health Organization MIS-C case definition (fever ≥3 days, elevated inflammatory markers and no other obvious microbial cause of inflammation, including bacterial sepsis, staphylococcal or streptococcal shock syndromes, and laboratory evidence of current or recent SARS-CoV-2 infection).^1^ (Table, Supplemental Digital Content 1, https://links.lww.com/INF/G623) Only hospitalized MIS-C patients were included in the study. Laboratory documentation included a positive nasopharyngeal, nasal or salivary polymerase chain reaction [PCR] or antigenic test, or a positive SARS-CoV-2 serology test. Children vaccinated against SARS-CoV-2 and those without consent were excluded.

Controls were drawn from a different source population than cases, the Vigil network (NCT0441231) in France across 74 ambulatory pediatric centers and 5 emergency units, and consisted of children 6 months to 15 years old, with no prior vaccination against SARS-CoV-2 and with laboratory-confirmed SARS-CoV-2 acute infection during the same calendar period, captured via a standardized electronic case report form completed by pediatricians.^18^ Laboratory documentation included a positive nasopharyngeal, nasal, or salivary PCR or antigenic test, or a positive nasal self-test. Controls vaccinated against SARS-CoV-2 were excluded.

### Exposure

The primary exposure was prior SARS-CoV-2 reinfection, defined as a documented previous infection ≥6 months (positive PCR, antigen test or serology) before the index date (MIS-C diagnosis for cases, infection date for controls). For controls, prior SARS-CoV-2 infection was ascertained through review of medical records from the Vigil network that documented prior infection. For MIS-C cases, parental report of earlier infection for cases was ascertained by structured telephone interview and used to supplement hospital record data, particularly where linkage to prior outpatient testing was limited.

### Outcome

The primary outcome was the difference in the proportion of SARS-CoV-2 reinfection (vs. primary infection) between MIS-C patients and their controls.

### Matching

A matched case–control design was chosen to address the potential confounding bias of our study related to the age, gender and epidemic period.

Each case was matched 1:1 to control on sex, epidemic period (by predominant national variant in France and Switzerland (Delta 21A [Period 1, from September to December 2021], Omicron 21K [Period 2, from January and February 2022], Omicron 21L [Period 3, from March and April 2022]) and age group (<25or ≥ 25 months).^19^ Random selection among eligible controls within each stratum was used. Broader age bands were chosen to maintain feasible matching and target sample size

### Variables and Data Sources

All diagnostic, clinical, microbiologic and outcome data for MIS-C were abstracted from hospital records. For cases, abstracted variables included sociodemographic characteristics; baseline health status; SARS-CoV-2 vaccination status; SARS-CoV-2 symptoms or contact with a SARS-CoV-2–positive patient; dates of admission; inflammatory markers (eg, erythrocyte sedimentation rate, C-reactive protein and procalcitonin); mucocutaneous signs; need for ventilatory or vasoactive support; cardiac dysfunction; coagulopathy; gastrointestinal symptoms; fever duration and laboratory evidence of SARS-CoV-2 infection. For controls, variables included age (months), sex, date of infection and laboratory evidence of SARS-CoV-2 infection. Data were captured in REDCap electronic case report forms per a prespecified protocol.

### Sample Size

Assuming a 10% prevalence of reinfection in the pediatric population and 3% among MIS-C cases, with 2-sided alpha of 0.05, 80% power and 1:1 matching ratio, the target sample size was 180 cases and 180 controls (total N = 360).

### Statistical Analysis

All data were analyzed using Python’s programming language (version 3.9.6 with the pandas, scipy, statsmodels, lifelines and numpy packages) in accordance with the original protocol and data protection guidelines. Two-sided *P* values <0.05 were considered statistically significant.

Categorical variables were reported as numbers (%); continuous variables as median (interquartile range [IQR]) and were compared using Student’s *t* tests, as appropriate.

### Primary Analysis

For primary analysis, the proportion of reinfection was compared within matched pairs using McNemar’s test, and matched odds ratios (ORs) were estimated, with Haldane–Anscombe correction when required.^20^

### Sensitivity Analysis

Three sensitivity analyses were performed.

First, conditional logistic regression for the matched pairs estimated the association between reinfection and MIS-C adjusted for sex (binary), age at disease onset (continuous variable in months) and epidemic period (categorical, based on dominant viral variants). Adjusted odds ratios (aORs) were calculated to estimate the effect of reinfection and potential confounders on the likelihood of MIS-C, ensuring that observed associations were not explained by these variables.

Second, an alternative 1:2 matching on age (continuous variable) re-estimated effects using McNemar’s test and matched OR, and a multivariate conditional logistic regression was performed with the same covariates.

Third, a multivariable logistic regression including all eligible controls assessed robustness to matching exclusions, adjusting for sex, age at the disease onset and epidemic period.

A complete-case approach was used for missing data.

### Ethical Considerations

In France, the RIPH 3 study protocol was approved by the French Personal Data Protection Committee (N°ID-RCB: 2023-A00031-44). In Switzerland, the research project was approved by Swiss Ethics (CER-VD and CER-GE: Nº2024-00918).

In France, for hospitalized MIS-C cases, letters were mailed to potential participants hospitalized for MIS-C between September 1, 2021, and April 30, 2022. Parents/legal guardians’ consent was obtained by telephone before inclusion.

In Switzerland, consent was obtained by signing a general consent form. Parents were contacted only to collect any data that may have been missing from the computerized files.

In the Vigil Study for the control patients, an information sheet was provided to all patients. Written consent was not required, but parents were informed, and data were analyzed unless parents expressed their refusal to participate.^18^

The study followed the STROBE reporting guideline for case–control studies and adhered to the Declaration of Helsinki.

Sponsors had no role in the design, conduct, analysis, interpretation or reporting of the study. The authors vouch for the accuracy and completeness of the data and for the conformity of the study to the protocol.

## RESULTS

### Study Population

The matched cohort included 180 children with MIS-C disease (median [IQR] age, 106 [62–136] months; 11 [6%] <25 months; 125 boys [69%] and 55 girls [31%]) (Fig. 1).

**FIGURE 1.**
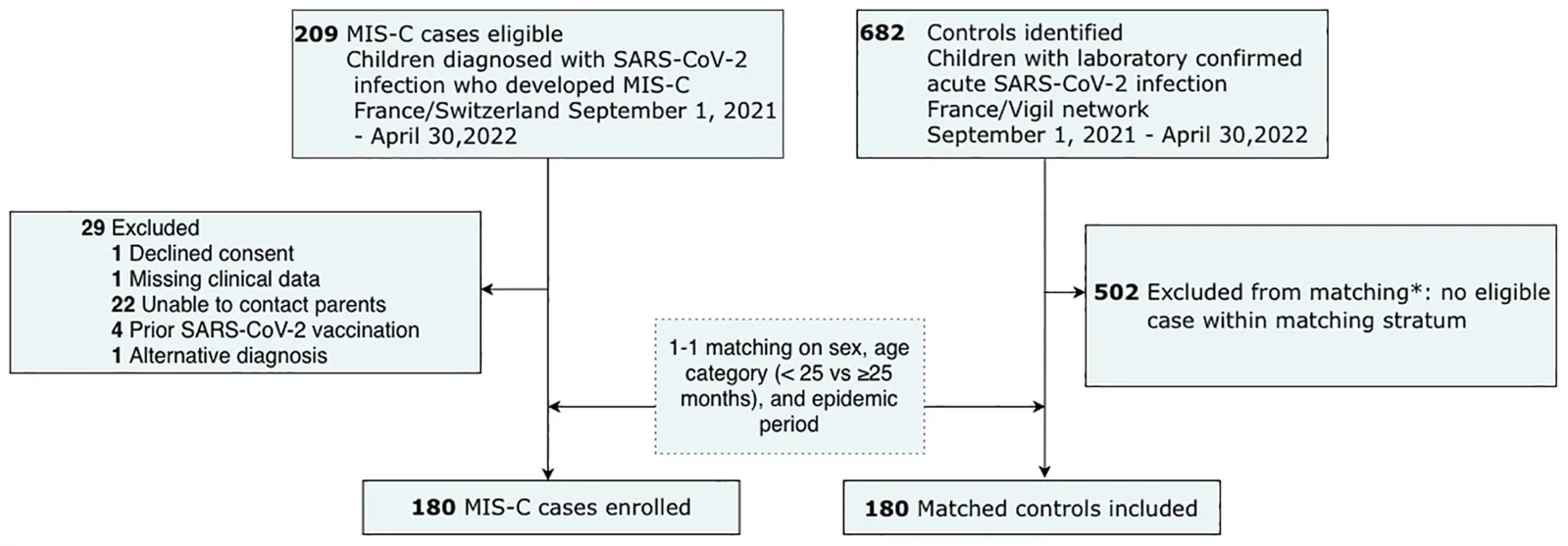
Study flow chart and matching profile. From 209 eligible MIS-C cases, 180 were enrolled. Of 682 potential controls, 180 were matched 1:1 on sex, age category and epidemic period. *Unmatched controls were not included in the primary matched analysis but were used in sensitivity analyses.

During September 1, 2021, to April 30, 2022, 682 controls with laboratory-confirmed SARS-CoV-2 infection were identified. After matching, 180 controls were included (median [IQR] age, 72 [48–108] months; 11 [6%] <25 months; 125 boys [69%] and 55 girls [31%]). Baseline demographics, clinical characteristics and laboratory findings for matched pairs are shown in Tables 1 and 2. Characteristics of all 682 controls identified before matching are presented in Table, Supplemental Digital Content 2, https://links.lww.com/INF/G623. Cases were older than controls (*P* < 0.001).

**TABLE 1.** Baseline Characteristics of Matched MIS-C Cases and Controls

Baseline Characteristics	Cases (n = 180)	Controls (n = 180)
Age at index date*, mo, median (IQR)	106 [62–136]	72 [48–108]
Age group, No. (%)		
<25 months	11 (6)	11(6)
≥25 months	169 (94)	169 (94)
Sex, No. (%)		
Male	125 (69)	125 (69)
Female	55 (31)	55 (31)
Country, No. (%)		
France	155 (86)	180 (100)
Switzerland	25 (14)	0 (0)
Epidemic period, No. (%)		
First: Sept–Dec 2021 (Delta 21A)	76 (42)	76 (42)
Second: Jan–Feb 2022 (Omicron 21K)	95 (53)	95 (53)
Third: Mar–Apr 2022 (Omicron 21L)	9 (5)	9 (5)
Any comorbidity†, No. %	34 (19)	NA
Obesity Other comorbidities	25 (14) 9 (5)	NA NA
MIS-C clinical features (cases only), No. (%)		
Mucocutaneous involvement‡	146 (81)	--
Hypotension or shock	47 (26)	--
Heart failure (clinical or laboratory evidence)§	138 (77)	--
Coagulopathy (laboratory evidence)	40 (22)	--
Acute gastrointestinal symptoms¶	136 (76)	--
Vasoactive support	33 (18)	--
Respiratory support∥	11 (6)	--

* “Index date” is MIS-C diagnosis for cases and infection date for controls. Pairs were matched 1:1 on sex, age category and epidemic period.

† Comorbidities included chronic respiratory disease, obesity (Obesity defined as BMI ≥95th percentile for age and sex.), chronic cardiac disease, chronic liver disease and diabetes.

‡ Mucocutaneous involvement included rash, conjunctivitis, oral cavity changes, swelling, and redness of hands and feet.

§ Echocardiographic evidence of Heart failure laboratory evidence included Left ventricular ejection fraction <55%, pericarditis or coronary dilation. Coronary dilatation defined by a *Z* score ≥2.5.

¶ Gastrointestinal manifestation included abdominal pain, vomiting and diarrhea.

∥ Respiratory support included intubation and noninvasive ventilation (BiPap and CPap).

BMI indicates body mass index; NA, not obtained.

Values are no. (%) unless otherwise indicated. Percentages have been rounded and may not total 100. — indicates not applicable.

**TABLE 2.** Virologic Characteristics of Matched MIS-C Cases and Controls

Characteristics	Cases (n = 180)	Controls (n = 180)
Prior SARS-CoV-2 reinfection, No. (%)	0 (0)	7 (4)
Recent close contact with SARS-CoV-2 positive person, No. (%)	38 (21)	128 (71)
SARS-CoV-2 testing modality*, No (%)		
Nasopharyngeal Antigenic testing	8 (4)	127 (71)
Positive nasopharyngeal SARS-CoV-2 RT-PCR	46 (26)	49 (27)
Positive antibody testing (serology)	126 (70)	0 (0)
Nasal self-antigenic test	0 (0)	71 (39)

* Testing modalities are not mutually exclusive; totals may exceed 100% because some participants had multiple positive tests.

RT-PCR indicates reverse transcriptase-PCR.

Unless otherwise indicated, data are expressed as No. (%) of participants. Percentages have been rounded and may not total 100.

By epidemic period, 76 (42%) index dates fell in period 1, 95 (53%) in period 2 and 9 (5%) in period 3. Laboratory confirmation was available for all patients. Serology and nasopharyngeal antigenic tests were the most frequently used modalities (each 70% in cases and 70% in controls).

### Primary Outcome

After matching, none of the MIS-C cases had a documented prior SARS-CoV-2 reinfection, whereas 7 of 180 (3.9%) did. The proportion of reinfections differed between groups (McNemar χ^2^ = 5.142; *P* = 0.023; matched OR, 0.067; 95% CI [0.004–1.167]; *P* = 0.064).

### Sensitivity Analysis

In conditional logistic regression for matched pairs, reinfection was associated with lower odds of MIS-C (aOR, 0.183; 95% CI [0.027–1.234]; *P* = 0.093), corresponding to an 81.7% reduction in odds (Table, Supplemental Digital Content 3, https://links.lww.com/INF/G623).

Reanalysis with 1:2 matching on age only yielded similar results (McNemar χ^2^= 7.111; matched OR, 0.053; 95% CI [0.003–0.906]; *P* = 0.008). Conditional logistic regression results are presented in Table, Supplemental Digital Content 4, https://links.lww.com/INF/G623.

An unmatched multivariable logistic regression including all eligible controls was directionally consistent (aOR, 0.146, 95% CI [0.017–1.287], *P* = 0.077) (Table, Supplemental Digital Content 5, https://links.lww.com/INF/G623).

## DISCUSSION

In this retrospective matched case–control analysis, none of the MIS-C cases had a documented prior SARS-CoV-2 reinfection, while 4% of matched controls did. Although the McNemar’s test reached statistical significance (McNemar χ^2^ = 5.142; *P* = 0.023), the matched-pairs OR and 95% CI (matched OR, 0.067; 95% CI [0.004–1.167]; *P* = 0.064) did not. These findings suggest a possible lower risk of MIS-C following reinfection.

These findings align with national and population-based data showing a sharp decline in MIS-C cases over successive pandemic waves.^14,21,22^ MIS-C per infection fell by 66% during the (post-vaccine) Delta period and by 95% during the Omicron period compared with Alpha (*P <* 0.001).^15^ In the United States, the Centers for Disease Control and Prevention surveillance documented a sustained decline, with only 117 MIS-C cases reported in 2023 and incidence 98% below the late-2020 peak.^23^ Consistent with milder pediatric disease in the Omicron wave, multicenter reports from Israel and US surveillance describe reduced severity indicators during Omicron compared with Alpha/Delta wave.^24,25^ The Omicron variant 21L in France and Switzerland, which became dominant from March 2022, has been associated with milder disease presentations in children, potentially explaining the decline in MIS-C cases over time.^16,26^ In this study, only 5% of the MIS-C cases occurred in this period, suggesting similar results.

Sensitivity analyses in our study were concordant with the primary result. A multivariable conditional model suggested 81.7% lower odds of MIS-C with reinfection versus primary infection (aOR, 0.183), although the estimate did not reach conventional statistical significance (*P* = 0.09), likely reflecting the small number of reinfections observed in the controls. For context, laboratory-based surveillance in France reported reinfections rising to 6.8% during Omicron, higher than the 4% seen among our matched controls.^16^ This could be plausibly explained by the younger age of our control cohort; therefore, a decreased chance of reinfection (median age 36 months; IQR [12–84]).

Several factors may underlie the reduced MIS-C risk at reinfection. One is increased population immunity.^3^Active immunity through vaccination against SARS-CoV-2 has been shown to drastically reduce MIS-C risk.^27,28^ In a US multicenter, test-negative design, 2 BNT162b2 doses conferred 91% effectiveness against MIS-C in adolescents, and French national data show a similar protective association in adolescents.^28,29^ Our results are in line with this hypothesis, as they suggest that the development of an immune passive response would protect children in the event of subsequent reinfection. The initial infection may trigger an exaggerated inflammatory or autoimmune response, leading to MIS-C in a subset of children. In contrast, reinfections may benefit from T- and B-cell memory responses, which help block early antigen dissemination and mitigate overactivation of the innate immune system.

By late 2022, there were significantly fewer MIS-C cases, and they have remained low ever since.^30^At the same time, serologic surveys indicated very high immunity levels in high-income settings, driven by vaccination, prior infection or both. In US blood donors, 96.4% had SARS-CoV-2 antibodies by Q3 2022.^31^A review summarizing the UK experience of SARS-CoV-2 in children stated that by September 2022, 98% of primary school-age children had a positive serology test for SARS-CoV-2.^32,33^Globally, a World Health Organization–-aligned meta-analysis also documented high seroprevalence by early 2022 across high-income regions.^34^ As SARS-CoV-2 transitions toward endemic circulation, one might expect that many children will acquire infection early in life—potentially in the first 5 years—and epidemiologic data suggest that MIS-C is less common in the youngest age groups compared with school-age children.^6,34^ The low rate of MIS-C cases since 2022 highlights the choice of a retrospective matched case–control study rather than a prospective study.

The temporal distribution of MIS-C in our cohort is consistent with the broader epidemiology of the syndrome, including the typical school-age peak and male predominance.^1–4^ Early US series reported a median age around 8 years and frequent gastrointestinal, cardiovascular and mucocutaneous involvement, features mirrored in our data.^4^ Indeed, in our study, the mean age of MIS-C occurrence was 106 months, and mostly boys were affected (69%). The main clinical features were mucocutaneous involvement (81%), heart failure symptoms or laboratory evidence (77%), and gastrointestinal symptoms (76%). Seventy percent of the MIS-C cases had a positive serology test. These findings align with other studies that show similar results.^13^

The study has several limitations. First, the case–control design precludes causal inference, though matched design and sensitivity analyses mitigate selection and confounding biases.

Second, the retrospective design may introduce recall bias, particularly in reporting prior infection. However, it is possible that the psychologic impact of MIS-C on families facilitated recall of whether it was a primary infection. Furthermore, we addressed this by using laboratory-confirmed reinfection from the prospective Vigil network for the controls.

Third, the study did not adjust for comorbidities and preexisting conditions, as there was missing information for the control patients. Among MIS-C cases, 19% had at least 1 comorbidity, with obesity being the most frequent (14%). The observation is consistent with the broader clinical literature, which demonstrates that MIS-C most frequently affects previously healthy children. Hoste et al show that apart from obesity, comorbidities are rare in MIS-C, with most cases presenting in otherwise healthy children.^35^ Because comparable data were not available for controls, we cannot exclude the possibility that differences in baseline health status between cases and controls may have influenced the observed associations in the study.

Fourth, cases were from Switzerland and France, whereas controls were from France only. However, epidemic waves and variant timing were similar across these countries during the study period, supporting comparability.^19^ Furthermore, controls were recruited from ambulatory pediatric centers and emergency units, whereas MIS-C cases were hospitalized at tertiary and university hospitals. This difference in recruitment settings may introduce selection bias, as the control population may not be fully representative of the source population from which cases arose. In particular, children followed in ambulatory care may have had more opportunities for outpatient SARS-CoV-2 testing, potentially increasing the likelihood of documented prior infections. Conversely, prior infections among MIS-C cases may have been under-documented if testing was not performed during earlier mild or asymptomatic infections. To mitigate this issue, prior infections in MIS-C cases were ascertained through both hospital records and structured parental interviews. Nevertheless, some degree of differential exposure ascertainment cannot be excluded.

Sixth, the small number of reinfections despite the use of a multicentric international study, including a large sample size and a nationwide design, limits the generalizability of our findings regarding reinfection and MIS-C risk. This finding may also be linked to the age difference between the 2 groups. The control group had a significantly smaller age group (mean age of 36 vs. 106 months, *P* < 0.001), and subsequently, a lower chance of reinfection. Further studies in other settings with larger cohorts and longer follow-up periods are needed to confirm these findings. Additionally, matching on broad age categories may allow residual confounding by age, as immune responses can differ substantially within age categories. Further studies could consider narrower age intervals to reduce potential confounding.

But to our knowledge, no other study evaluated the risk of MIS-C following subsequent SARS-CoV-2 infection. Additionally, this study highlights important clinical and public health implications, suggesting the protective role of natural immunity against severe postinfectious complications.

## CONCLUSIONS

This study indicates that MIS-C most commonly occurs after primary SARS-CoV-2 infection. Evidence for a reduced risk following reinfection is suggestive but not conclusive, given the CIs and limited statistical significance in sensitivity analyses. While the retrospective case–control design has limitations, the findings align with previous research showing a decline in MIS-C cases over time and across pandemic waves. With MIS-C incidence now low but nonzero, ongoing vaccination and surveillance remain essential to prevent severe postinfectious complications and to detect shifts in risk should new variants emerge.

## ACKNOWLEDGMENTS

*We thank Dr Camille Jung, Mathilde Servera, Pr Fabrice Michel, Lydia Slimani, Samira Plassart and Berenice Lecardonnel.*

## Supplementary Material
