# Supplementary material for: SARS-CoV-2 Reinfection and Risk of Multisystem Inflammatory Syndrome in Children: A Case–control Study
Source: Pediatr Infect Dis J. 2026 May 20;45(11):986–91. doi: 10.1097/INF.0000000000005278 (PMC13649262; doi:10.1097/INF.0000000000005278)
Supplement: Supplementary file 1 [file inf-45-0986-s001.pdf]

SUPPLEMENTAL DIGITAL CONTENT 1: WHO criteria for MIS-C included the following(1):

Children and adolescents 0–19 years old with fever > 3 days
AND two of the following:
Rash or bilateral non-purulent conjunctivitis or muco-cutaneous inflammation signs (oral, hands or feet)
Hypotension or shock
Features of myocardial dysfunction, pericarditis, valvulitis, or coronary abnormalities (including echocardiographic findings or elevated Troponin/NT-proBNP)
Evidence of coagulopathy (by prothrombin time, partial thromboplastin time, elevated D-Dimer levels)
Acute gastrointestinal symptoms (diarrhea, vomiting, or abdominal pain)
AND elevated markers of inflammation such as erythrocyte sedimentation rate, C-reactive protein level, or procalcitonin level
AND no other obvious microbial cause of inflammation, including bacterial sepsis, staphylococcal or streptococcal shock syndromes
AND evidence of COVID-19 (RT-PCR-, antigen test- or serology-positive), or likely contact with patients with COVID-19

SUPPLEMENTAL DIGITAL CONTENT 2. Demographic and clinical characteristics of the 682 control patients

Baseline characteristics	Controls (n=682)
Age at index date[1], mo, median (IQR)	36 [12-84]
Age group, No. (%)	
< 25 months	307 (45)
≥ 25 months	375 (55)
Sex, No. (%)	
Male	361 (53)
Female	321 (47)
Country, No. (%)	
France	682 (100)
Switzerland	0 (0)
Epidemic period, No. (%)	
1st: Sept-Dec 2021 (Delta 21A)	131 (19)
2nd: Jan-Feb 2022 (Omicron 21K)	388 (57)
3rd: Mar-Apr 2022 (Omicron 21L)	163 (24)
Prior SARS-CoV-2 reinfection, No. (%)	12 (1.7)
Recent close contact with SARS-COV-2 positive person, No. (%)	495 (73)
SARS-CoV-2 testing modality* [2]*, No (%)	
Nasopharyngeal Antigenic testing	518 (76)
Positive SARS-CoV-2 RT-PCR	117 (17)
Positive antibody testing (serology)	0 (0)
Nasal self-antigenic test	201 (29)

Unless otherwise indicated, data are expressed as No. (%) of participants. Percentages have been rounded and may not total 100. [1] : "Index date" is infection date for controls. [2]: Testing modalities are not mutually exclusive; totals may exceed 100% because some participants had multiple positive tests.

Abbreviations : RT-PCR : reverse transcriptase-PCR.

SUPPLEMENTAL DIGITAL CONTENT 3. Conditional logistic regression for matched pairs

Variable	aOR [95% CI]	P-value
**Age (mo)**		
0 mo	Reference	
Increase 1mo	1.017 [1.011,1.023]	<.001
**Prior SARS COV-2 reinfection**		
No	Reference	
Yes	0.183 [0.027,1.234]	.093

C-statistic: 0.66

All the sensitivity analyses were conducted using multiple imputation by chained equations, except for the McNemar analysis. This approach was required because no reinfections were observed in the case group, leading to a structural zero that precluded standard comparative analyses. By imputing reinfection status for a small subset of patients, we were able to explore the robustness of our findings under alternative plausible scenarios. In that analysis, we simulated an alternative scenario by randomly selecting 20 case patients and assigning them a history of SARS-CoV-2 reinfection. The analysis including the matching patients in a 1:1 ratio (matching on age, gender, and epidemic period) and in a 1:2 ratio (matching only on age) was performed using a conditional logistic regression model. The analysis that included the entire study population was performed using a standard multivariable logistic regression model. The analysis assessed the independent association between SARS-CoV-2 reinfection and MIS-C, controlling for potential confounders, sex, age, and epidemic period. The adjusted odds ratios (aOR), 95% confidence intervals (CI), and P-values are presented in Tables, Supplemental Digital Content 3, 4 and 5.

SUPPLEMENTAL DIGITAL CONTENT 4. Conditional logistic regression with 1:2 matching on age

Variable	aOR [95% CI]	P-value
**Age (mo)**		
0 mo	Reference	
Increase 1mo	1.014 [1.009,1.020]	<.001
**Sex**		
Female	Reference	
Male	1.767 [1.157, 2.698]	.007
**Epidemic period**		
Previous period	Reference	
Actual period	0.508 [0.374,0.690]	<.001
**Prior SARS COV-2 reinfection**		
No	Reference	
Yes	0.232 [0.033,1.646]	.148

C-statistic: 0.63

SUPPLEMENTAL DIGITAL CONTENT 5. Multivariable logistic regression analysis including all eligible controls showing predictors of developing a MIS-C

Variable	aOR [95% CI]	P-value
**Age (mo)**		
0 mo	Reference	
Increase 1mo	1.021 [1.017,1.025]	<.001
**Sex**		
Female	Reference	
Male	1.769 [1.184,2.644]	.004
**Epidemic period**		
Previous period	Reference	
Actual period	0.455 [0.332,0.623]	<.001
**Prior SARS COV-2 reinfection**		
No	Reference	
Yes	0.146 [0.017,1.287]	.077

C-statistic: 0.87
